# Rapid and simple detection of endospore counts in probiotic *Bacillus* cultures using dipicolinic acid (DPA) as a marker

**DOI:** 10.1186/s13568-018-0633-0

**Published:** 2018-06-19

**Authors:** Xiao-Sheng Liang, Chun Liu, Zhu Long, Xiao-Hua Guo

**Affiliations:** Provincial Key Laboratory for Protection and Application of Special Plants in Wuling Area of China, College of Life Science, South-Central University for Nationalities, No. 182, Minyuan Road, Hongshan District, Wuhan, 430074 Hubei China

**Keywords:** *Bacillus*, Probiotics, Endospore detection, Dipicolinic acid, Fluorescence

## Abstract

Spore counting in probiotic *Bacillus* cultures using dipicolinic acid (DPA) as a marker was studied for developing a rapid and simple detection method. The newly developed method is based on the fluorescence enhancement by a new chelating agent, which forms a complex with EuCl_3_ and DPA. The results showed that 1,2-cyclohexanediamine-N,N,N′N′-tetraacetic acid (CyDTA) greatly enhanced the fluorescence intensity in all selected chelating agents. The optimal composition of the fluorescence complex DPA-Eu-CyDTA had a detection limit of 0.3 nM of DPA. Metal ions in high concentrations, including Cu^2+^, Fe^2+^, Fe^3+^, Al^3+^, and Zn^2+^, might lower the detection sensitivity, which could be eliminated by diluting the sample with the metal ions below 10 μM. The maximum release of DPA was achieved by heating treatments at 121 °C for at least 10 min for two types of *Bacillus* endospores. The spore concentrations and corresponding released DPA fluorescence intensities were linearly associated (coefficient *R*^2^ = 0.9993 and 0.9995 for *Bacillus subtilis* MA139 and *Bacillus licheniformis* BL20386, respectively). The detection limit for both strains reached approximately 6800 spores/mL. The verification results showed that the DPA fluorimetry assay developed in the present study was fully consistent with the plate-counting assay. The study shows that the fluorescence complex DPA-Eu-CyDTA can be reliably used for the detection of endospores in *Bacillus* fermentation for the production of probiotics.

## Introduction

*Bacillus* species have been extensively studied for use as probiotics during the past 20 years both for scientific exploration and commercial development (Hong et al. 2005; Cutting 2011). Spore probiotics are widely used in humans for medical purposes as dietary supplements for preventing diarrhea and intestinal disorders. The strain of *Bacillus licheniformis* BL20386 used in this study is the main ingredient in probiotic Zhengchangsheng^®^ capsule, a useful therapeutic agent for the treatment of diarrhea (Heo et al. 2014). In the field of animal nutrition, spore probiotics enhance the growth performance and disease-resistance of poultry, swine, and shrimps (Cutting 2011). The *B. subtilis* MA139 strain, also used in this study, has been applied as feed probiotics, benefiting piglets’ growth and modifying intestinal microflora (Guo et al. 2006). Except for non-spore *Lactobacillus* probiotics, endospores are extremely heat stable and acid resistant, which enables long-time storage without loss of viability during storage at room temperature and survival when passing through the gastric barrier.

In the production of probiotics from spore formers of *Bacillus*, the growth and accumulation of spores are carefully monitored to ensure a high fermentation yield (Zhao et al. 2008; Khardziani et al. 2017). To lower the cost of high spore production, maximum spore yields are often optimized before scale-up fermentation (Pryor et al. 2007; Chen et al. 2010). In any situation, spore counts must be accurately quantified to control the fermentation. The routine method for spore quantification often relies on plate-counting assays, which generally are a time-consuming and quite tedious method (Hazan et al. 2012). In recent years, numerous researchers have studied the development of rapid methods for spore detection that can be partial alternatives to routinely monitoring spores (Hindle and Hall 1999; He et al. 2003; Fichtel et al. 2007; Postollec et al. 2010; Bai et al. 2014; Hebert et al. 2017). Many analytical techniques have been based on the detection of dipicolinic acid (DPA) in endospores as a unique constituent, including high-performance liquid chromatography (HPLC), photofluorescence, and capillary zone electrophoresis (Hindle and Hall 1999; Pellegrino et al. 2002; Fichtel et al. 2007; Ai et al. 2009). With its tridentate ligand structures, DPA can specifically form a complex with terbium (Tb), one of the lanthanides, and exhibit enhanced luminescence when irradiated by UV light. This method has a limit of detection (LOD) of 2 nM of DPA (Hindle and Hall 1999). Combined with the technique for DPA released from endospores, the reported LOD for the Tb-DPA luminescence method reached 10^3^–10^4^ *B. subtilis* spores per mL (Hindle and Hall 1999; Pellegrino et al. 2002). However, the LOD for DPA based on Tb-DPA fluorescence often depends on the concentration of reagents (Hindle and Hall 1999), which might cause trouble in the simultaneous detection of different samples with various DPA contents. Meanwhile, excess Tb in the complex produces high background fluorescence and leads to a lower LOD in the detection (Hindle and Hall 1999; Ai et al. 2009).

In the present study, another DPA-Eu—based luminescence method was explored for simple detection and a lower LOD for DPA. Two strains, *B. subtilis* MA139 and *B*. *licheniformis* BL20386, were used as models to assess the endospore counts based on a DPA fluorimetry assay, which was studied for monitoring and optimizing spore production in probiotics commercialization.

## Materials and methods

### DPA fluorimetry assay

DPA standards with a purity of 99% were purchased from the Aladdin Company (Cat. No. P109609; Shanghai, China). A standard stock solution of DPA with a 10 mM concentration was prepared by dissolving 161.72 mg of DPA in 100 mL of Tris–HCl buffer (50 mM, pH 8.0; the same below). The stock solution was stored at 4 °C and diluted by Tris–HCl buffer to produce working solutions at different concentrations before use. Two common lanthanide ions, EuCl_3_ and TbCl_3_ (Alfa Aesar, 99.9% purity), were selected for possible fluorescence enhancement when they chelated with DPA in the presence of chelating agents. Two mM of EuCl_3_ and TbCl_3_ were prepared in Tris–HCl buffer. As structural analogues, four chelating agents were screened for fluorescence enhancement, including ethylenediaminetetraacetic acid (EDTA), 1,2-cyclohexanediamine-N,N,N′N′-tetraacetic acid (CyDTA), 2-hydroxyethylethylenediamine triacetic acid (HEDTA), and ethylene glycol tetraacetic acid (EGTA). The chelating agents were prepared in Tris–HCl buffer in 2 mM concentrations. A lanthanide solution was first mixed with an equal volume of a chelating agent solution, and then a 0.4 mL DPA sample was added to the premixed, 3.6 mL lanthanide-chelating agent solution to produce the fluorescence complex in a final volume of 4 mL. The fluorescence intensity was detected by a Hitachi F-7000 spectrofluorophotometer (Hitachi Ltd., Tokyo, Japan). The pre-set parameters were a scanning speed of 3000 nm/min, a 10 nm slit, 700 V of photo-multiplier tube (PMT) voltage for Eu-based complexes and 400 V for Tb-based complexes, and a responding time of 0.08 s. Each time, the fluorescence complexes were freshly prepared and then detected immediately. First, the fluorescence complexes with different chelating agents were scanned from 500 to 700 nm at a 270 nm excitation wavelength. The 1.8 mM lanthanide solutions (equal to the same concentration of lanthanides in the fluorescence complexes) were also scanned to detect possible fluorescence intensity. As a control, a mixture of 0.4 mL DPA sample, 1.8 mL lanthanide solution, and 1.8 mL of Tris–HCl was also scanned for a fluorescence peak. Tris–HCl buffer was used as a blank control. The chelating agent that produced the highest fluorescence peak was selected for DPA detection. The fluorescence complex with the selected chelating agent was further scanned to determine the excitation and emission wavelengths based on the DPA standard in a used concentration.

The linear correlation of the fluorescence intensity coupled with the DPA concentration was tested based on three parallel experiments. In order to improve the accuracy of measurements, the DPA samples were diluted to adjust the light output in arbitrary units on a scale from 0 to 1000 arbitrary units (AU) based on earlier reports (Hindle and Hall 1999; Fichtel et al. 2007).

### Metal ions interference

The effect of metal salts on the fluorescence intensity was studied for possible interference. Ten metal ions (NaCl, CaCl_2_, KCl, MgSO_4_, MnSO_4_, CuSO_4_, FeSO_4_, FeCl_3_, AlCl_3_, and ZnSO_4_) were dissolved in a 4 μM DPA solution to reach the concentrations of 10, 1, 0.1, and 0 mM, respectively. Then, a 0.4 mL DPA solution containing a metal ion was mixed with 2 mL of EuCl_3_ and 2 mL of CyDTA to produce fluorescence complexes. The fluorescence intensity of the mixtures containing different ions was then measured. The fluorescence intensity was compared with the control based on three replications.

### Bacterial strain and culture media

Two representative probiotic *Bacillus* strains, *B. subtilis* MA139 and *B*. *licheniformis* BL20386, were used in the study. *B. subtilis* MA139 was deposited in the China General Microbiological Culture Collection Center (CGMCC) as CGMCC 7.90. *B*. *licheniformis* BL20386 was obtained from the China National Center for Medical Culture Collections (CMCC) as CMCC 63516. The strains were kept at − 80 °C in 20% sterile glycerol before use. Luria–Bertani (LB) media (10 g/L tryptone, 5 g/L yeast extract, and 10 g/L NaCl; pH 7.0) was used for the strains’ cultivation and fermentation. The strains were cultured in rotating shaker at 37 °C and 200 rpm.

### Preparation of endospores and spore suspensions

The strains were grown and sporulated in nutrient sporulation medium (NSM) (Vasantha and Freese 1979) for 48–60 h at 37 °C. The mature spores were harvested after a heat shock of 80 °C for 15 min. The spores were purified by washing twice and then were resuspended in Tris–HCl buffer with an OD_600 nm_ of about 1.0 (equivalent to 10^8^ spores/mL). Each time the spores were freshly prepared for the experiment.

### DPA release from endospores

Freshly prepared spore suspensions were diluted by Tris–HCl buffer to a concentration of approximately 10^7^ colony-forming units per mL (CFU/mL). Then, the dilution was treated immediately for the release of DPA from endospores. The two methods used were heat shock and l-alanine—triggered germination (Pellegrino et al. 2002). As for the heating shock, the spore suspensions were autoclaved in screw-cap glass test tubes at 105 °C for 15 min, 115 °C for 20 min, 121 °C for 10 min, and 121 °C for 20 min, respectively. After cooling, the supernatants containing the DPA were collected after centrifugation (2500×*g* for 10 min) and tested for fluorescence intensity. In the other method, the freshly prepared spores were centrifuged and suspended in 100 mM l-Ala solution (in Tris–HCl buffer). Every 1, 2, and 3 h, the suspensions were sampled for the detection of fluorescence intensity. All the experiments were conducted with three replications.

### Spore plate-counting assay

The traditional plate-counting assay was used to determine the spore concentrations (Chen et al. 2010). Spores were first heated at 80 °C for 15 min to kill vegetative cells, and then the spore suspensions were serially diluted in physiological saline. The spore dilutions were plated on an LB agar medium, and the colonies were counted after cultivation at 37 °C for 24 h. The results were expressed as CFU/mL.

### Validation of spore counting

The spore suspensions of the two strains, each of which had an initial OD_600 nm_ of 1.0, were treated by autoclaving, and then the supernatants were collected by centrifugation (2500×*g* for 10 min). The supernatants were then serially diluted twofold, and the corresponding DPA fluorescence intensity of the two strains was detected. The initial concentrations of the two spore suspensions were separately detected by plate-counting assays. Correspondingly, a linear correlation between spore concentrations (CFU/mL) and the fluorescence intensity (AU) was built for the two strains.

The verification experiments for the detection of spore concentration in *Bacillus* fermentation were carried out by sampling the fermented broths in LB media from the two strains at 12, 24, and 36 h. The samples were washed twice and suspended in an equal volume of Tris–HCl buffer. The spores were then quantified by a fluorimetry assay and a plate-counting assay. The obtained results were expressed as the means of fluorescence intensity or CFU/mL and their standard deviation (SD) based on three replicated experiments. The spore concentrations based on the fluorimetry assay were further converted to CFU/mL according to the standard curves of the two strains. The Student’s *t* test in JMP 11.0 (SAS Institute Inc., USA) was used to test the differences between the two assays. A *P* value less than 0.05 was regarded as a significant difference.

## Results

### DPA standard fluorimetry assay

**Fig. 1:**
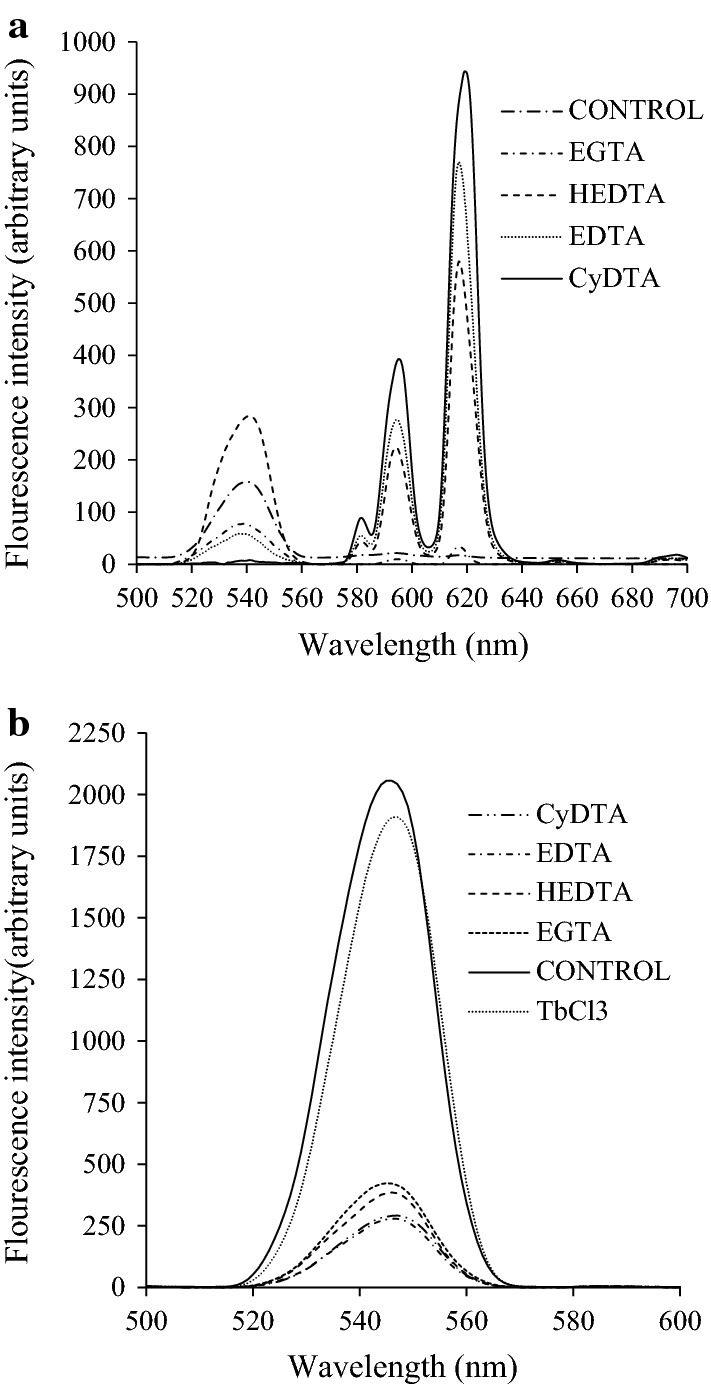
Effects of different clathrates with EuCl_3_ (**a**) and TbCl_3_ (**b**) on DPA fluorescence intensity and emission fluorescence spectra. DPA final concentration 400 nM (**a**), and 100 nM (**b**)

The supplementation of chelating agents in DPA-Eu complexes greatly enhanced the production of fluorescence signals by EGTA, HEDTA, EDTA, and CyDTA (Fig. 1a). The supplementation of CyDTA showed the best effect in improving the fluorescence intensity compared with the other three chelating agents. No fluorescence signal was detected in the DPA-Eu complex. The TbCl_3_ showed high background fluorescence, and the supplementation of chelating agents greatly decreased the fluorescence intensity compared with the Tb-DPA complex (Fig. 1b). The 1.8 mM TbCl_3_ complex produced a fluorescence intensity of 1847 AU at 546 nm. Chelated with 100 nM DPA, the fluorescence intensity of the Tb-DPA complex only increased to 2057 AU.

**Fig. 2:**
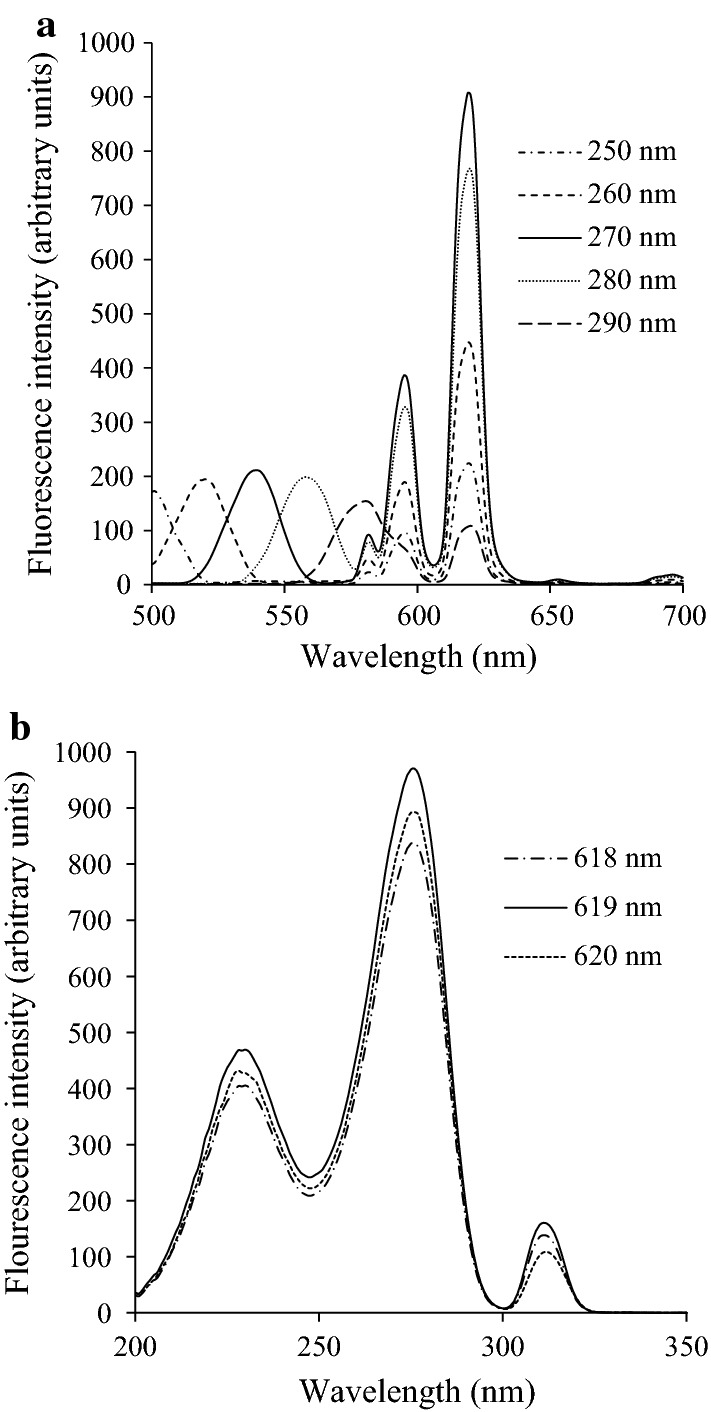
Emission (**a**) and excitation (**b**) fluorescence spectra of DPA-Eu-CyDTA with the DPA final concentration of 400 nM

**Fig. 3:**
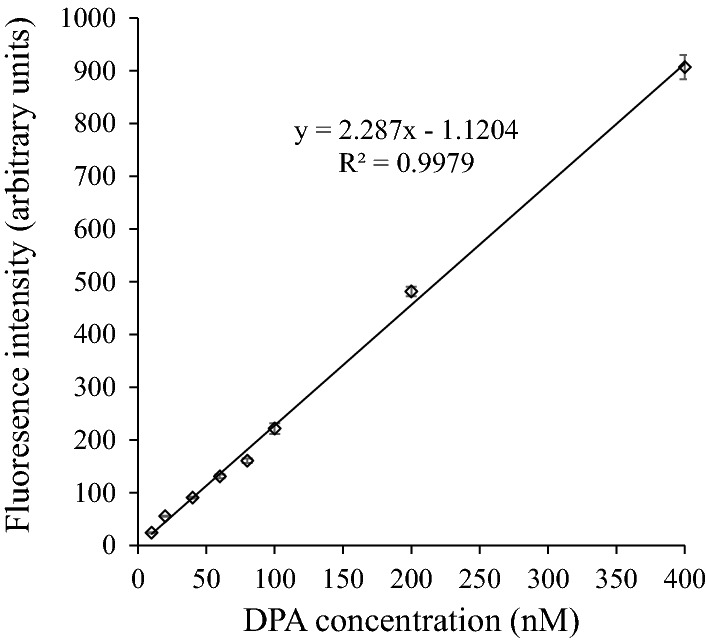
The standard curve of DPA ranging from 10 to 400 nM

The fluorescence complex DPA-Eu-CyDTA was further studied for specific DPA detection. The peak height was greatly affected by the excitation light, and the highest peak was observed at the excitation wavelength of 270 nm (Fig. 2a) and emission wavelength of 619 nm (Fig. 2b). Under the optimal excitation and emission wavelengths, the fluorescence intensity of the complex was proportional to the DPA concentration over a range from 10 to 400 nM with a higher correlation coefficient (*R*^2^ = 0.9979; Fig. 3). The LOD for DPA was 0.3 nM based on the formula LOD = 3*σ*/*S*, where *σ* is the SD of the background fluorescence with ten repetitions and *S* is the slope of the calibration curve (Rai and Rai 2008).

### Effect of metal ions on DPA fluorimetry assay

**Fig. 4:**
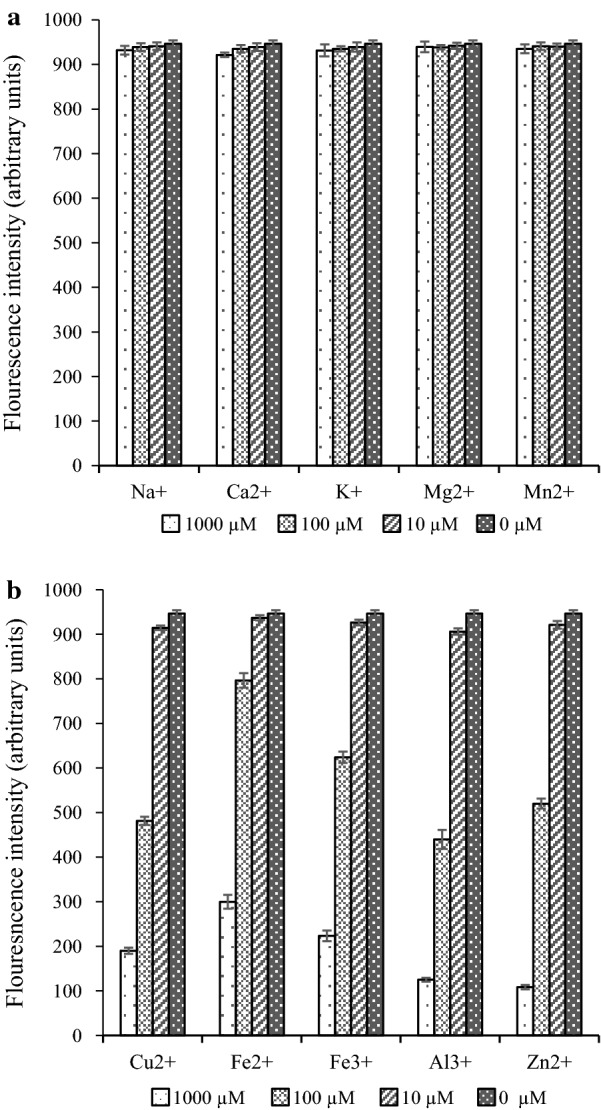
The effect of different metal ions (**a** Na^+^, Ca^2+^, K^+^, Mg^2+^, Mn^2+^; **b** Cu^2+^, Fe^2+^, Fe^3+^, Al^3+^, Zn^2+^) at different concentrations (1000, 100, 10 and 0 μM) on the DPA fluorescence intensity

In all, ten metal ions were tested in the study. The inclusion of Na, Ca, K, Mg, and Mn had no effect on the fluorescence intensity of the DPA-Eu-CyDTA complex based on the present study (Fig. 4a). Some other metal ions, including Cu^2+^, Fe^2+^, Fe^3+^, Al^3+^, and Zn^2+^, might lower detection sensitivity based on the results in Fig. 4b. However, even with the presence of those ions in the DPA solutions, the concentration of ions could be diluted to be under 10 μM to eliminate the interference.

### Effect of treating methods on DPA release from endospores

**Fig. 5:**
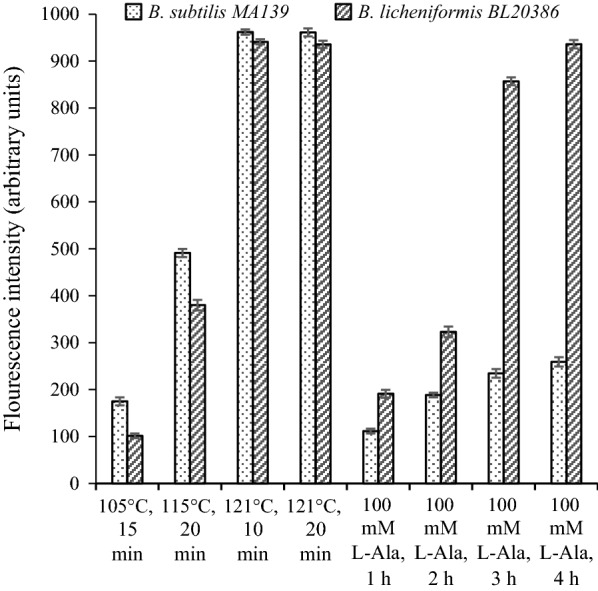
Effect of different treatments on the release of DPA from endospores of *B. subtilis* MA139 and *B*. *licheniformis* BL20386. The value of fluorescence intensity is based the 30-fold dilution of the spore suspensions with OD_600nm_ = 1.0

The maximum release of DPA was achieved by heating treatments at 121 °C for at least 10 min for both *Bacillus* spores (Fig. 5). In this study, *B. subtilis* MA139 was almost non-responsive to the l-Ala method since only about 25% of the DPA was released after 3 h of culturing with l-Ala compared to the heating treatment at 121 °C for 20 min. The endospores of *B. licheniformis* BL20386 were more sensitive to l-Ala, and more than 90% of the DPA was released when treated by 100 mM of l-Ala for 3 h. The full release of DPA was achieved after treatment by 100 mM of l-Ala for 4 h. In terms of the speed and efficiency of DPA release in the two current methods, the heating treatment was preferable for spore quantification, and the treatment at 121 °C for 10 min was enough for the full release of DPA for the two strains.

### Calibration curves of spore counts based on DPA fluorimetry assay

**Fig. 6:**
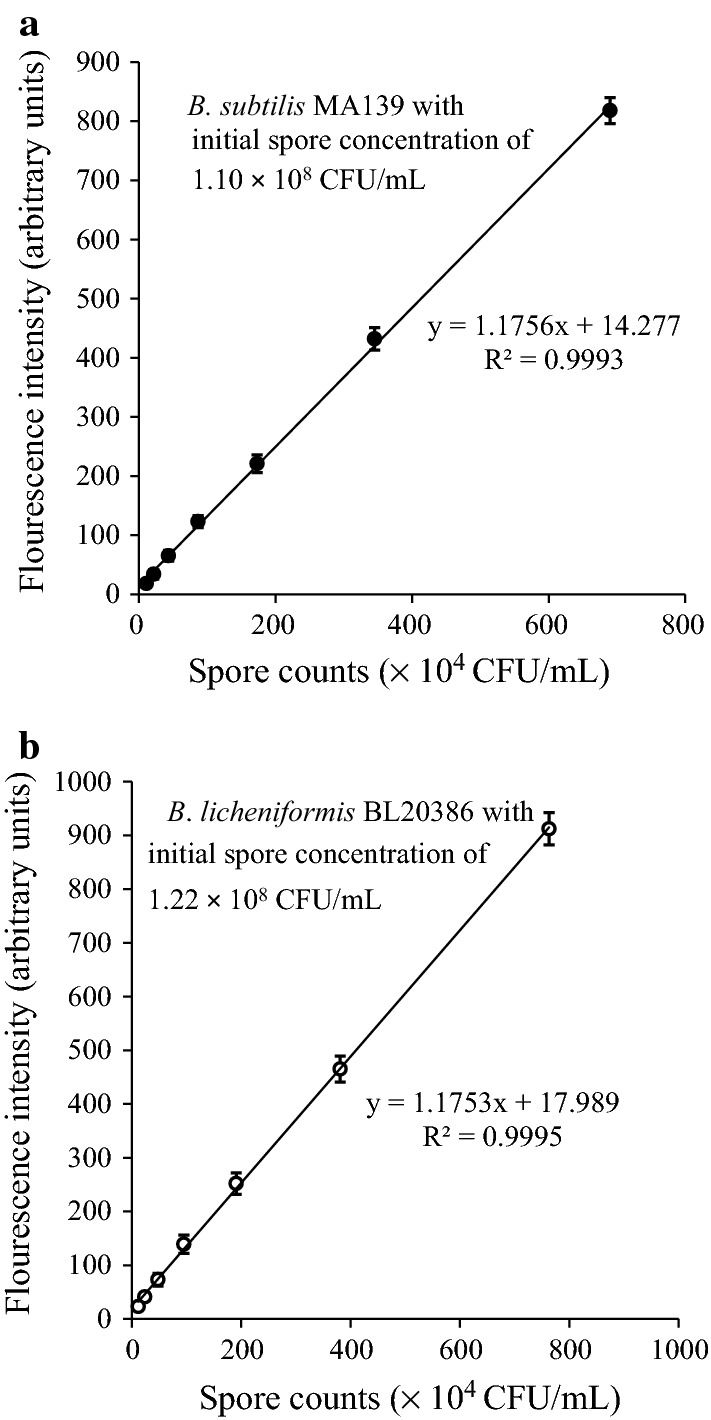
Calibration curves of spore counts of *B. subtilis* MA139 (**a**) and *B*. *licheniformis* BL20386 (**b**) and their fluorescence intensity

Figure 6 shows a good linear correlation between the spore concentrations and corresponding released DPA fluorescence intensity (coefficient *R*^2^ = 0.9993 and 0.9995 for *B. subtilis* MA139 and *B. licheniformis* BL20386, respectively). The LOD for the both strains reached approximately 6800 spores/mL.

### Verification of spore counting in fermented broth

The results of spore detection based on DPA fluorimetry and the plate-counting assay are separately presented in Table 1. At each sampling point, no significant differences between the two assays were found for the two *Bacillus* strains (*P* > 0.05).

**Table 1:** The confirmative results on spore detection by DPA fluorimetry assay of *B. subtilis* MA139 and *B. licheniformis* BL20386 compared with Plate counting assay

Sampling time (h)	*B. subtilis* MA139 concentration (CFU/mL)		*B*. *licheniformis* BL20386 concentration (CFU/mL)	
	DPA fluorimetry assay	Plate counting assay	DPA fluorimetry assay	Plate counting assay
12	(4.35 ± 0.21) × 10^4^	(4.05 ± 0.35) × 10^4^	(5.85 ± 0.63) × 10^4^	(5.55 ± 0.78) × 10^4^
24	(5.65 ± 0.21) × 10^5^	(5.50 ± 0.36) × 10^5^	(8.30 ± 0.28) × 10^5^	(7.90 ± 1.27) × 10^5^
36	(4.32 ± 0.11) × 10^7^	(3.80 ± 0.35) × 10^7^	(4.70 ± 0.14) × 10^7^	(4.30 ± 1.27) × 10^7^

## Discussion

Lanthanide ions chelated by DPA show enhanced fluorescence by energy transference. Their unique fluorescent characteristic is often adapted for the determination of DPA from spore-formers in environmental samples (Fichtel et al. 2007). The Tb-based DPA fluorimetry assay has been intensively studied for determining the number of endospores since the DPA-Tb complex shows the advantage of highly enhanced fluorescence and a long fluorescence lifetime (Rosen et al. 1997; Hindle and Hall 1999; Pellegrino et al. 2002). However, in the study by Hindle and Hall (1999), the desired LOD for DPA often relied on excess Tb compared with DPA, and different concentrations of Tb could result in different calibration curves corresponding to standard DPA concentrations. Moreover, unchelated Tb could produce too high background fluorescence (Hindle and Hall 1999), which was in line with the result in Fig. 1b. Therefore, the LOD may depend on desirable concentrations of TbCl_3_, and the concentrations of reagents are often adjusted before detection (Hindle and Hall 1999). However, in fermentation for spore production, the spore concentration often relies on the culture time and fermentation conditions, and many analytes with different spore concentrations produced in spore optimization designs must be detected simultaneously (Ren et al. 2018). Therefore, the DPA-Tb fluorimetry assay cannot be used as a general analytical method for rapid endospore quantification.

Alternatively, Ai et al. (2009) reported a method based on Eu fluorescence nanoparticle sensor for the possible detection of *B. anthracis* spores. Using Eu-based fluorescence with a red emission offers the advantages of a higher penetrating power and a higher resistance to interferences, which particularly benefit the detection of spores in complex environments. The direct excitation of DPA-Eu complexes produces a weak fluorescent signal, and the background fluorescence can be negligible (Hindle and Hall 1999). Hindle and Hall’s results were in accordance with the findings in Fig. 1a. EDTA was often used as a chelating agent for fluorescence enhancement (Arnaud and Georges 2003; Ai et al. 2009; Reisfeld et al. 2011). However, in the present study, the complex of DPA-Eu-CyDTA exhibited significantly higher fluorescence intensity compared with that of Eu-EDTA-DPA. This is probably associated with the higher hydrophobicity in the molecular structure of CyDTA than that of EDTA, which lowers the nonradiative quenching of the Eu emission caused by high-frequency OH oscillations of water molecules (Ai et al. 2009). This is the first report on fluorescence enhancement by CyDTA in a DPA-Eu complex. The DPA-Eu-CyDTA fluorescence complex provided an excellent linear correlation with DPA concentration with an LOD of 0.3 nM (Fig. 3). The LOD of DPA is close to the value reported by Ai et al. (2009) with 0.2 nM. However, the method in this study is simpler, and the fluorescent complex is readily prepared by just mixing the DPA samples, Eu, and CyDTA in the correct proportion. The method is especially suitable for the circumstances of the spore detection in fermentation optimization and quality control and also for the simultaneous detection of endospores in a large number of samples (Ren et al. 2018).

The DPA in bacterial spores exists in the form of a CaDPA complex. Therefore, the release of DPA from endospores could also be caused by the addition of Ca^2+^ in the solution. Fortunately, the presence of Ca^2+^ showed no interference, even with a high concentration of 1 mM. The interference of other ions on fluorescence detection depends on the ion concentrations. The interference can be eliminated if the concentration is diluted to less than 10 μM. In practical samples from spore fermentation, the spore suspensions are often treated by washing and above 100-fold dilutions (Ren et al. 2018). The possible metal ions are then greatly diluted, and their interference is likely to be negligible.

l-Ala has often been the primary germinant to induce spores’ germination and the release of DPA into the solution (Setlow 2003). The present results showed that the bacterial endospores of different species had diverse responses to the same germinants (Fig. 5), which was also verified in Vanderberg’s report (2000). However, conducting a heating treatment for at least 10 min led to the full release of DPA for both strains. In the report by Pellegrino, all available DPA was extracted by autoclaving at 121 °C for 30 min. The difference in treatment time might be associated with the ramp time in different experimental conditions and the specific strains. In consideration of the difference of endospores, a slightly longer autoclave treatment was suggested to ensure the full release of DPA (Pellegrino et al. 2002). In terms of the two currently used methods’ speed and efficiency of DPA release, the heating treatment appears to preferable for spore quantification. In the present study, the LOD of approximately 6800 CFU/mL for both strains was close to the level in the earlier reports by Pellegrino (10^3^ CFU/mL) and Alistair (10^4^ CFU/mL). According to the verification experiment shown in Table 1, the results indicated that the spore count detected by the fluorimetry assay was completely consistent with the plate-counting assay.

In conclusion, this study provided a rapid and simple detection method for quantifying *Bacillus* probiotics endospores based on a new chelating agent to form the fluorescence complex DPA-Eu-CyDTA. The optimal system in the fluorescence complex DPA-Eu-CyDTA had a detection limit of 0.3 nM of DPA. The spore concentrations and correspondingly released DPA fluorescence intensity were linearly correlated, and the LOD for the both *Bacillus* strains reached approximately 6800 spores/mL. The method developed in this study was verified to be efficient in the detection of endospores in *Bacillus* fermentation for probiotics production.

## Abbreviations

AU: arbitrary units
CFU: colony-forming units
CGMCC: China General Microbiological Culture Collection Center
CMCC: China National Center for Medical Culture Collections
CyDTA: cyclohexanediamine tetraacetic acid
DPA: dipicolinic acid
EDTA: ethylenediaminetetraacetic acid
EGTA: ethylene glycol tetraacetic acid
Eu: europium
HEDTA: 2-hydroxyethylethylenediamine triacetic acid
HPLC: high performance liquid chromatography
l-Ala: l-alanine
LB: Luria–Bertani
LOD: limit of detection
NSM: nutrient sporulation medium
OD: optical density
PMT: photomultiplier tube
Tb: terbium
UV: ultraviolet
SD: standard deviation
